# Supplementary material for: Field Research on Mixing Aeration in a Drinking Water Reservoir: Performance and Microbial Community Structure
Source: Int J Environ Res Public Health. 2019 Oct 31;16(21):4221. doi: 10.3390/ijerph16214221 (PMC6862099; doi:10.3390/ijerph16214221)
Supplement: Supplementary file 1 [file ijerph-16-04221-s001.docx]

Supplementary Materials

**Figure S1.** Vertical distribution of water temperature during the running of water lifting aeration system (9.28 means 28th September; 10.1 means 1st October).

**Figure S2.** Vertical distribution of dissolved oxygen during the running of water lifting aeration system.

**Figure S3.** Distribution of main phyla in enhanced areas during the operation of water lifting aeraters (The vertical axis represents the percentage of different bacteria; X-Y-Z, area-period-location; X = C and E mean control and enhanced area；Y = 1, 2, 3, 4, mean 2018-09-28, 2018-10-15, 2018-10-29, 2018-11-28; Z = S, M, B, mean surface, medium and bottom water layer).

**Figure S4.** Changes in *Proteobacteria* in the enhanced area during operation of the water-lift aeration system. (The labeling is consistent with Figure S3.).
